# Improving ultrasound images with elevational angular compounding based on acoustic refraction

**DOI:** 10.1038/s41598-020-75092-8

**Published:** 2020-10-23

**Authors:** Parastoo Afshari, Christian Zakian, Vasilis Ntziachristos

**Affiliations:** 1 Chair of Biological Imaging, Technical University of Munich, 81675 Munich, Germany; 2 Institute of Biological and Medical Imaging, Helmholtz Zentrum München, 85764 Neuherberg, Germany

**Keywords:** Biotechnology, Engineering

## Abstract

Ultrasound imaging is affected by coherent noise or speckle, which reduces contrast and overall image quality and degrades the diagnostic precision of the collected images. Elevational angular compounding (EAC) is an attractive means of addressing this limitation, since it reduces speckle noise while operating in real-time. However, current EAC implementations rely on mechanically rotating a one-dimensional (1D) transducer array or electronically beam steering of two-dimensional (2D) arrays to provide different elevational imaging angles, which increases the size and cost of the systems. Here we present a novel EAC implementation based on a 1D array, which does not necessitate mechanically rotating the transducer. The proposed refraction-based elevational angular compounding technique (REACT) instead utilizes a translating cylindrical acoustic lens that steers the ultrasound beam along the elevational direction. Applying REACT to investigate phantoms and excised tissue samples demonstrated superior suppression of ultrasound speckle noise compared to previous EAC methods, with up to a two-fold improvement in signal- and contrast-to-noise ratios. The effects of elevational angular width on speckle reduction was further investigated to determine the appropriate conditions for applying EAC. This study introduces acoustic refractive elements as potential low cost solutions to noise reduction, which could be integrated into current medical ultrasound devices.

## Introduction

Speckle noise is an inherent property of ultrasound (US) imaging that results from constructive and destructive interference of backscattered acoustic waves caused by heterogeneities in tissue^1–4^. While speckle can be exploited to obtain dynamic information (e.g. on blood flow)^5^, it can also degrade both the resolution and contrast of static structures and blur the boundaries of layered tissues, which can hinder the interpretation of tissue morphology and fine structure and adversely affect diagnostic procedures^6–10^. Image post-processing or compounding methods are commonly used to reduce speckle in US imaging^11–20^. Post-processing is based on image filter algorithms that use information extracted from the images^11–13^, which limits realistic enhancement of structures obscured by speckle noise. Compounding methods average sequential images from the same field of view (FOV) with varied spatial or frequency content^7–10^, enhancing correlated features while removing uncorrelated speckle noise, which can reveal structures obscured by speckle in individual images. Frequency compounding methods either vary the emitted frequency or decompose the spectrum of the echo signal to obtain images with uncorrelated speckle patterns, while spatial compounding methods acquire images at different US beam orientations^14–20^. However, both compounding methods typically result in a loss of spatial or temporal resolution, as capturing multiple images reduces acquisition speeds and collecting signals from adjacent fields of view reduces the lateral resolution^18^.

Elevational angular compounding (EAC) is a type of spatial compounding that can simultaneously offer high speckle noise reduction and good temporal resolution, making it desirable for medical applications^19^. EAC obtains partially correlated images by steering the elevational imaging plane with small angular steps^19,20^. EAC methods typically employ either one-dimensional (1D) arrays that can only control the US beam in the azimuthal direction (i.e. parallel to the imaging plane) or two-dimensional (2D) arrays that can control the US beam in both the azimuthal and elevational directions (the latter being perpendicular to the imaging plane)^21–23^. US systems with 1D arrays are simple and inexpensive. However, implementing EAC in such systems necessitates bulky mechanical stages to physically move or rotate the 1D array in order to steer the beam in the elevational direction^19^, which is impractical in clinical applications. In contrast, US systems integrating 2D arrays can electronically steer the beam along the elevational direction without changing the detector’s position^21–23^.However, EAC has only been preliminarily validated in systems integrating 2D detector arrays on simulated data and, although this implementation was reported in patents^24,25^, it has not thus far been experimentally validated. Furthermore, the use of 2D array detectors increases the size and cost of the US system. Thus, there is a need for a means of incorporating EAC into US systems that are both economical and have a small form factor.

In this work, we aimed to develop a method of implementing EAC, which could be integrated into simple and low cost US system without sacrificing the image enhancement capabilities. We hypothesized that an acoustic refractive lens could steer a US beam from a 1D transducer array, imparting it with the elevational steering ability of a stationary 2D array while retaining the advantages in size, cost, and simplicity of a 1D array. Furthermore, linear micro-translation of a refractive element should impart precision control of elevational angular steering in a 1D transducer array while minimizing motion artefacts compared to rotating the entire transducer. We describe herein this refraction-based EAC technique (REACT) and assess its qualitative and quantitative enhancements of US images in experiments on phantoms and tissues ex vivo. Moreover, we examine the effect of experimental parameters that can cause image deformation due to compounding different elevational angular views.

## Methods

### Image acquisition

**Figure 1:**
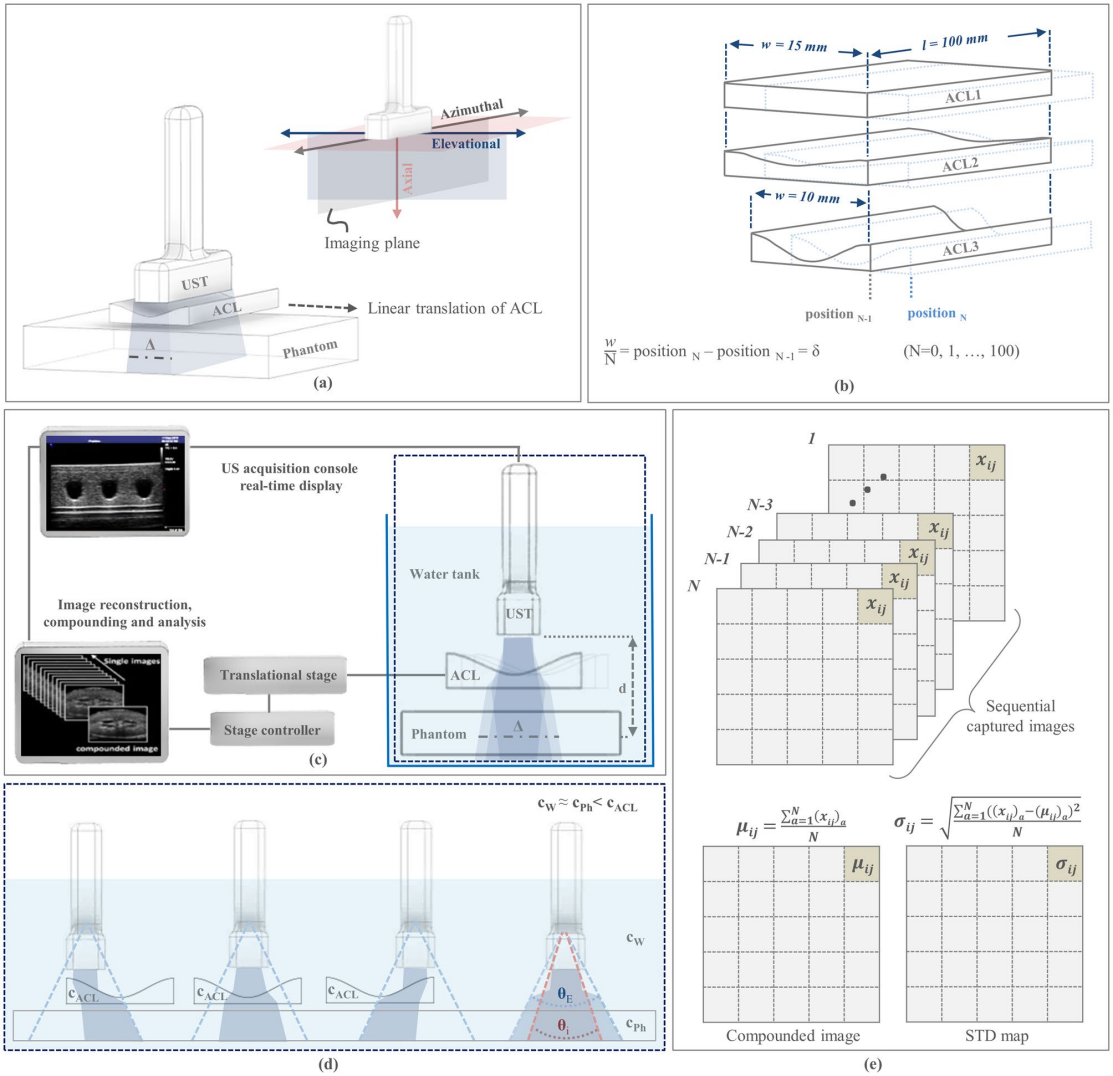
REACT elevational angular steering concept and implementation. (**a**) Linear translation of the ACL along the elevational direction in front of the stationary 1D transducer controls the elevational angular FOV. UST, ultrasound transducer, ACL, acoustic cylindrical lens, Δ, elevational angular width. (**b**) Renderings of ACL1, ACL2, and ACL3, which produce 0°, 2.5° and 5° angular deflections, respectively. The width and length of the ACLs are indicated by *w* and *l*, respectively. The ACLs are moved in consistent step sizes (δ), which are equal to the width of the ACL (w) divided by number of images (N) needed for compounding. (**c**) REACT imaging acquisition configuration. d, imaging depth. (**d**) Elevational angular steering in different positions of the ACL. Sound waves propagate through the water, phantom, and ACLs at speeds of c_w_, c_Ph_, and c_ACL_, respectively, where c_w_ ≈ c_Ph_ < c_ACL_. Imaging at different positions of the ACL extends the elevational angular FOV (θ_E_) of the transducer compared to its inherent elevational angular FOV (θ_i_). (**e**) Matrix operators used to compute the compounded image and standard deviation (STD) decorrelation map at the pixel level using the sequentially captured images.

The implementation scheme of REACT using an acoustic refractive element is shown in Fig. 1a,c. In short, linear translation of the acoustic cylindrical lens (ACL) across the stationary 1D transducer array steers the acoustic beam along the elevation direction and can be adjusted by changing the ACL’s position and radius of curvature (Fig. 1b). A linear transducer array with 128 unfocused elements and a central frequency of 7.5 MHz (12L5V, Terason, USA) connected to a portable acquisition console (Terason 2000 + , USA) was employed for US imaging. A motorized translation stage (MTS50-Z8, Thorlabs) was used to shift the ACL at predetermined linear steps (δ) in front of the 1D array transducer to obtain different elevational angular views by virtue of acoustic refraction. Each step has an approximate error of 0.7% of the step size. Because the lens translation was not automated, the acquisition was limited to an average of 20 frames per minute. However, implementing REACT with automated lens translation would allow for frame rates that are only limited by the acquisition speed of the US imaging system. Moreover, to provide two different effective elevational angular widths (Δ) needed for image fidelity exploration, samples were imaged at two different imaging depths (d). Figure 1d depicts the elevational steering angle of the acoustic beam at different positions of the ACL.

### Refractive element fabrication

Customized ACLs designed to cover the full sensor area were manufactured from Polymethyl methacrylate (PMMA), which has a sound speed of 2750 m/s and low acoustic attenuation of 1.4 dB/cm/MHz^26,27^. The lenses were fabricated from 2.5 mm ± 4 µm thick PMMA blocks. Figure 1b shows a schematic of the three manufactured lenses, ACL1, ACL2, and ACL3, with infinite (flat), 88 mm, and 24 mm curvature radii, respectively. The acoustic beam was steered in the elevational direction by refraction caused by the difference in acoustic impedance (Z) between the PMMA lens (Z = 3.23 × 10^6^ kg/m^2^s) and water (the imaging medium, Z = 1.49 × 10^6^ kg/m^2^s)^26,27^. The distance between the transducer and ACL was held to < 1 mm (while avoiding contact) to minimize the effect of multiple reflection artefacts on the image due to the impedance mismatch between the PMMA and water. Each ACL can produce a characteristic maximum elevational angular deflection, which is 0° for ACL1, 2.5° for ACL2, and 5° for ACL3. Given the radius of curvature, the usable scanning length (w) is geometrically constrained and the translation step is defined to acquire a fixed number of images (N = 100). ACL1 and ACL2 were scanned with steps of δ_1_ = δ_2_ = 150 µm, whereas ACL3 was scanned in steps of δ_3_ = 100 µm. The total deflection angle was theoretically calculated using Snell’s law as c_1_sinθ_2_ = c_2_sinθ_1_ (where c_1_ and c_2_ are the longitudinal wave velocities, and θ_1_ and θ_2_ are incidence and exit angles in materials 1 and 2, respectively)^28,29^ and confirmed experimentally as follows.

### Effective field of view and elevation angle characterisation

The transducer’s inherent elevational angular FOV (θ_i_), without ACLs, was first determined by measuring the distance between two opposing needle tips, which were inserted into opposite sides of an agar phantom until they just appeared on each side of the image. By recording their imaging depth, an inherent elevation angular FOV of 29.5° was calculated for the employed linear transducer. Similarly, the extended elevational angular FOVs (θ_E_) when employing ACL2 and ACL3 were calculated as 32° and 34.5°, respectively. The effective compounding angles of 0° (ACL1, serving as a non-angular compounding reference), 2.5° (ACL2), and 5° (ACL3) were computed by subtracting the inherent transducer angular elevation FOV from the extended ones obtained for each ACL.

### Imaging samples

Custom acoustic phantoms (50 mm × 20 mm × 15 mm) comprising 2% agar and 4% TiO_2_ in 100 ml water were manufactured to assess speckle reduction efficiency and image fidelity. Phantom A embedded three 7 mm-diameter cylindrical holes and was used to evaluate REACT’s speckle reduction efficiency. Phantom B contained three different holes in the shape of a cylinder (no diameter gradient), frustum (intermediate diameter gradient), and cone (high diameter gradient) and was used to explore the effect of elevational angular width and the target’s cross-sectional variation on EAC image fidelity. An excised chicken heart and swine kidney were utilized as biological phantoms to further explore the importance of the cross-sectional appearance of the sample on image fidelity for a given elevational angular width using REACT. The chicken heart was chosen due to its conical shape to represent a sample with high cross-sectional variation. The swine kidney, which is larger than a chicken heart, was selected because of its low cross-sectional variation. For comparability, US images of the biological phantoms were captured and despeckled under the same imaging condition utilizing ACL3. The phantoms were stabilized during imaging by pinning them to polystyrene foam. No live specimens were used in the experiments.

### Analysis method

Sequential images were captured in different positions of the ACL and compounded using a mean compound operator^30^ (Fig. 1e). The intensity and standard deviation (STD) were mapped at the pixel-level to investigate the efficiency of REACT to generate correlated structures and uncorrelated speckle patterns across acquired images (Fig. 1e). Single and compounded images were compared for each elevational angular deflection case to assess the despeckling efficiency. Rectangular regions of interest (ROIs = 28 × 140 pixels) within solid and empty (holes) regions in the phantom were selected to derive the intensity average and standard deviation to compute SNR and CNR. These indices were used as quantitative indicators of image improvement. The speckle and electrical noise suppression was also evaluated by inspecting the A-line intensity profiles of the single and compounded images.

## Results

**Figure 2:**
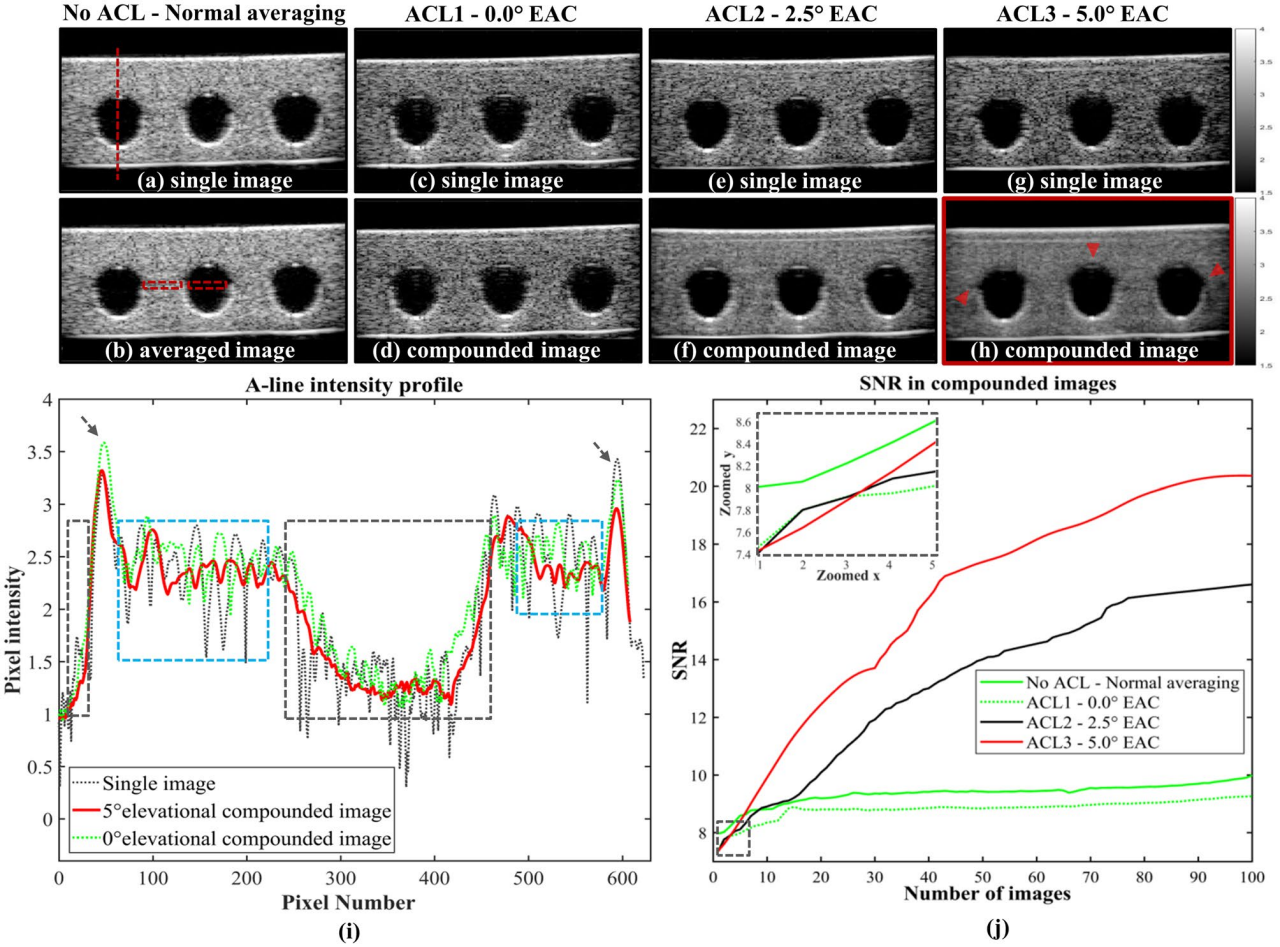
Speckle reduction achieved with REACT. (**a**)**–**(**h**) Single and compounded US images of a phantom with embedded cylindrical holes (phantom A) using three different ACLs. Images captured through no ACL in (**a**) and (**b**)**,** ACL1 (0° deflection angle) in (**c**) and (**d**), ACL2 (2.5° deflection angle) in (**e**) and (**f**), and ACL3 (5° deflection angle) in (**g**) and (**h**). Arrows in (**h**) show despeckled features and preserved edges obtained with the 5° elevational compounding. (**i**) A-line intensity profiles along the vertical dashed line in (**a**) applied through the single and compounded images with 0° and 5° elevation angle deflection; arrows indicate the phantom’s surfaces. Gray rectangles indicate water-filled regions above the top surface and inside the hole, blue rectangles indicate the regions inside the solid phantom. (**j**) Effect of increasing the number of images on SNR for the cases of normal averaging, using no ACL, and compounding, using ACL1, ACL2, and ACL3. Rectangular ROIs in (**b**) show regions used for deriving the SNR and CNR in (j) and Table 1.

Figure 2 shows the speckle reduction efficiency achieved using REACT. Figure 2a–h depict uncompounded (single), averaged and compounded US images of a phantom with cylindrical holes (phantom A) at direct view and elevational angular deflections of 0°, 2.5°, and 5°.Visual inspection of the US image pairs in Fig. 2a–h reveals noticeable speckle noise reduction with wider elevational angular deflection, with the greatest despeckling effect observed for the 5° case. Despite this wide deflection angle (5°), the edges of the holes in the phantom are preserved upon compounding, as highlighted by the arrows in Fig. 2h. Figure 2i shows the A-line intensity profiles obtained for a single image (Fig. 2c) compared to profiles of the images that were compounded at elevational deflections of 0° (Fig. 2d) and 5° (Fig. 2h). Inspecting the A-line intensity profile of the single image across the solid phantom sections (blue rectangles in Fig. 2i) shows high intensity variations related to the granular nature of speckle noise. These variations are strongly suppressed by REACT at an elevational angular deflection of 5°. In contrast, compounding with a 0° elevational angular deflection, which acts as a comparative reference to normal averaging, only moderately suppresses the intensity variations. The noise reduction upon compounding at 0° is primarily due to electrical noise averaging, which is apparent in the A-line intensity profile variation across the water-filled regions (gray rectangles in Fig. 2i), where no scatterers are expected to produce speckle. Still, even in the absence of speckle, compounding at a 5° elevational angle results in greater noise reduction than at 0°, demonstrating that REACT reduces both speckle noise and overall US electrical noise more efficiently than normal averaging. To quantify this observation, Fig. 2j shows the effect of increasing the number of images used for averaging and compounding on the SNR, as calculated within the ROI (red rectangle) in the solid region of the phantom in Fig. 2b. As expected, SNR increases for all cases, yet at a greater rate for the 5° EAC case, owing to a reduction in both speckle and electrical noise. Note that, for all EAC cases (with the ACL), initial SNR is lower compared to the normal averaging case (without the ACL), as seen in the inset of Fig. 2j. This is due to the signal loss caused by the acoustic attenuation and reflection. However, owing to the high despeckling efficiency of REACT, the final SNR in the 5° EAC case is two times higher than for normal averaging (Fig. 2j).

Table 1 shows SNR and CNR improvements obtained using REACT. Quantitative indices extracted from the two ROIs (28 × 140 pixels) specified in Fig. 2b were used to compare the despeckling efficiency of normal averaging and REACT at different elevational angular deflections. The higher SNR and CNR in the wider elevational angular acquisition are consistent with the observations in Fig. 2.

**Table 1:** SNR and CNR improvement using REACT. SNR: signal to noise ratio = µ/σ, CNR: contrast to noise ratio =|µ_Hole_ − µ_Phantom_|/σ_Hole_.

ACL Type	Phantom (SNR_compounded_ /SNR_single_ − 1)	Phantom and Hole (CNR_compounded_/CNR_single_ − 1)
No ACL (normal averaging)	0.24	0.27
ACL1 (0.0° deflection angle)	0.23	0.28
ACL2 (2.5° deflection angle)	1.22	1.84
ACL3 (5.0° deflection angle)	1.72	2.18

**Figure 3:**
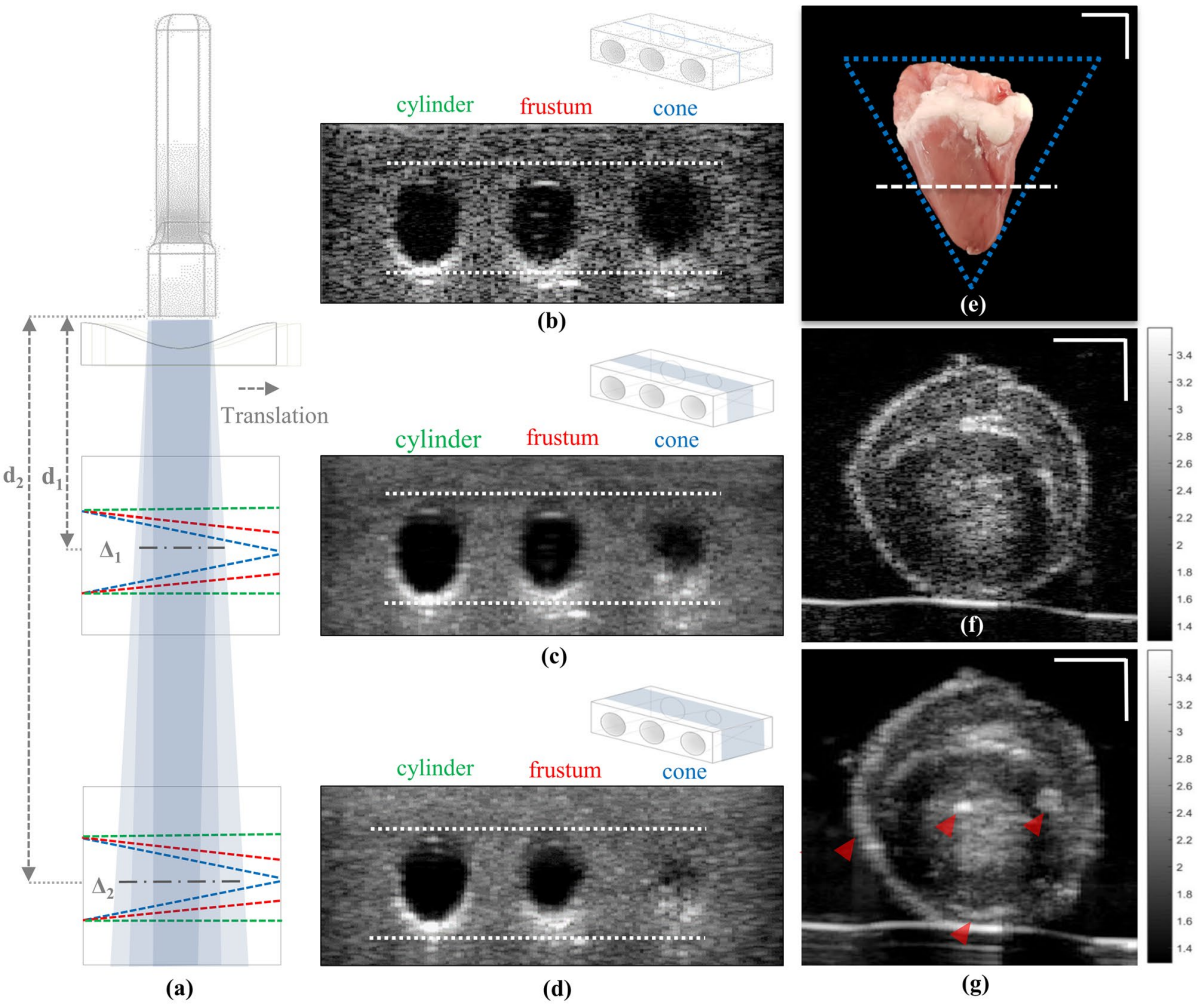
Elevational angular width affects elevational angular compounded image fidelity. (**a**) Schematic of the imaging acquisition configuration depicting the elevation angular width (Δ_1_≃1.3 mm and Δ_2_≃4.8 mm) at different phantom distance positions (d_1_ = 1.5 cm and d_2_ = 5.5 cm). Green, red, and blue dashed lines show the cross-section of the cylinder, frustum, and conical hole shapes in the phantom, respectively (phantom B). (**b**) Single US image of the phantom at depth d_2_. White dashed lines show the holes’ boundaries. (**c**), (**d**) 5° elevational compounded images at depths d_1_ and d_2_, respectively. (**e**) Excised chicken heart; white dashed line shows the image cross section location in (**f**) and (**g**). Blue dashed lines indicate the conical shape of the chicken heart. (**f**), (**g**) Single and 5° elevational compounded US images of chicken heart at depth d_2_. Red arrows highlight the effect of compounding and loss of fidelity compared to the single image in (**f**). Scale bars: 1 mm in e, 0.5 mm in (**f**) and (**g**).

Figure 3 demonstrates the effect of elevational angular width (Δ) and the target’s cross-sectional variation on image fidelity after elevational angular compounding. The schematic in Fig. 3a illustrates the configuration of phantom B, which contains three holes with shapes approximating a cylinder, frustum, and cone. These holes have different diameter gradients along the entire angular imaging width, enabling the investigation of the correlation between Δ and image fidelity. Phantom B was imaged using ACL3 at two different imaging depth (d_1_ = 1.5 cm and d_2_ = 5.5 cm), i.e. two effective Δ values. A single image and two compounded images using 5° elevational angular deflection are shown in Fig. 3b–d. As expected, image fidelity upon compounding suffered most for the conical hole (Fig. 3c,d, cone), which has the highest diameter gradient along elevational direction, even for the shallow imaging depth (d_1_, smaller Δ). The cone with an intermediate diameter gradient (Fig. 3c,d, frustum) displays acceptable image fidelity for the smaller Δ at d_1_, but is more impacted at d_2_. However, the cylindrical hole (no diameter gradient, labelled cylinder) shows image fidelity for both Δ cases, without blurry edges. The impacts of these findings were further explored using a biological phantom of an excised chicken heart (Fig. 3e). Figures 3f,g show the single (Fig. 3f) and compounded (Fig. 3g) images obtained from the organ with the ACL3 at d_2_. Visual examination of these US images reveal edge blurring and structure distortion in the compounded image (arrows in Fig. 3g) due to the high diameter gradient of the organ in the elevational direction, similar to the cone case in Phantom B (as depicted by the blue dotted triangle in Fig. 3e).

**Figure 4:**
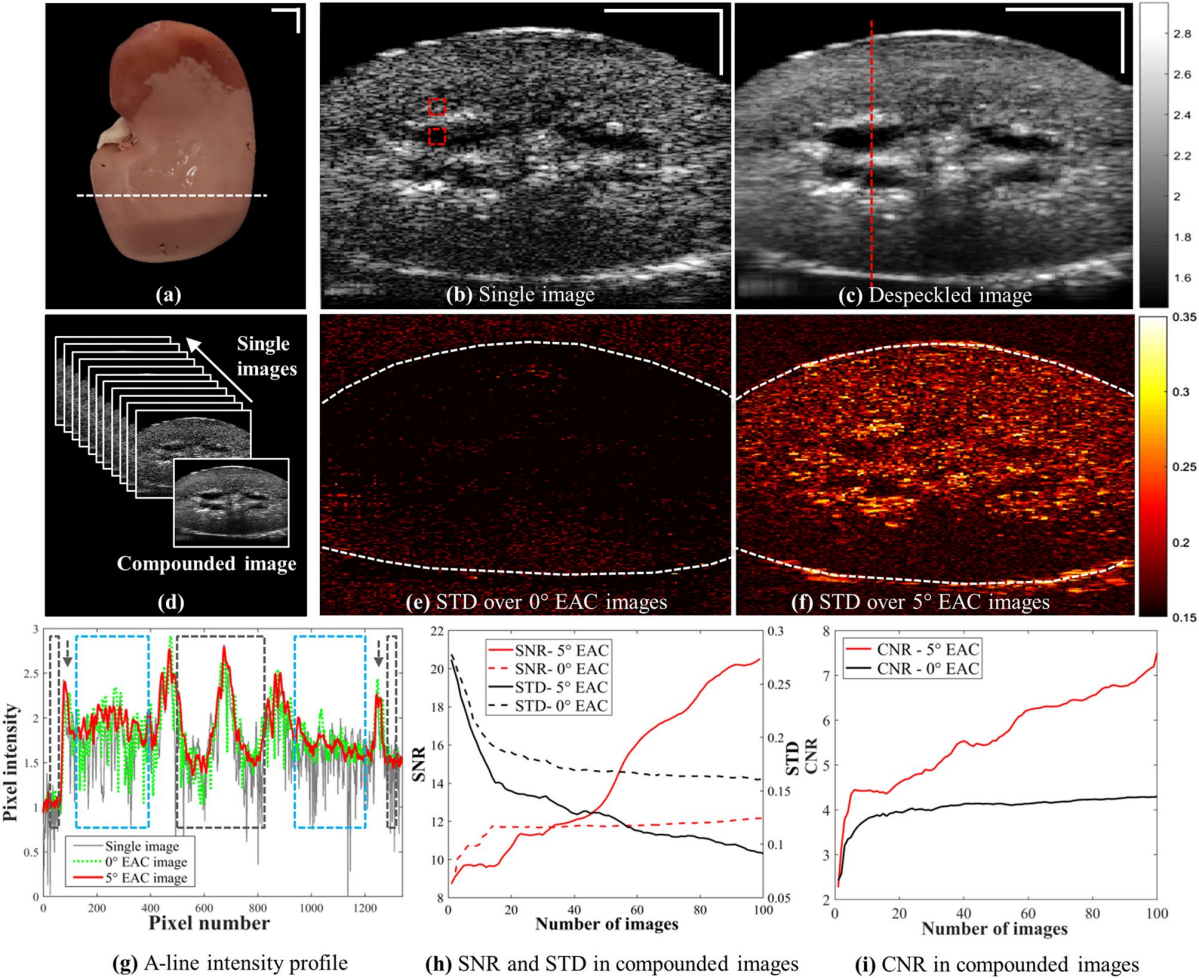
REACT demonstrates efficient US speckle reduction in a swine kidney. (**a**) Excised swine kidney; white dashed line shows the image cross-section location in (**b**,**c**). (**b**)**, **(**c**) Single and compounded US images of a swine kidney at a depth of 5.5 cm using ACL3. Rectangular ROIs in (**b**) show regions used for deriving the SNR, STD and CNR indices plotted in (**h**) and (**i**). (**d**) Representation of the compounding of a series of images by REACT. (**e**)**, **(**f**) Intensity of STD maps at pixel-level along all captured images, using ACL1 and ACL3, respectively; dashed lines delineate the tissue surface contour. (**g**) A-line intensity profiles along the vertical dashed line in (**c**) applied through the single and elevational compounded images within 0° and 5° elevation angle deflection. Arrows demonstrate the tissue’s surfaces. Grey rectangles indicate hypoechoic regions above the top surface and inside the holes. Blue rectangles indicate regions within the tissue. Scale bars: 1 mm. (**h**) Effect of increasing the number of images used for compounding on SNR and STD within tissue and inside the hole for the cases of 0° and 5° EAC. (**i**) Effect of increasing the number of images used for compounding on CNR for the cases of 0° and 5° EAC.

Figure 4 demonstrates speckle reduction upon applying REACT to image a swine kidney (Fig. 4a), which has a cylindrical shape and thus a low diameter gradient. Figure 4b,c show single and compounded US images of the swine kidney, with the latter produced using ACL3 at a depth of d_2_ = 5.5 cm. Visual inspection of the US images in Fig. 4 reveals marked speckle noise reduction using REACT without blurriness or structure distortion, despite the use of the same imaging conditions as for the chicken heart (Fig. 3g). The difference in image fidelity between the two organs is attributable to the lower variation of the swine kidney’s cross-sectional appearance (low diameter gradient) across the angular imaging width compared to that of the chicken heart. Figure 4e,f display the variation in pixel intensity for images compounded at elevational angles of 0° and 5°, which was mapped to investigate the speckle pattern decorrelation between individual captured images (Fig. 4d). As expected, higher speckle pattern decorrelation is obtained for images captured within the 5° elevational angle, which therefore resulted in higher speckle reduction as demonstrated in Fig. 4c.

Figure 4g shows A-line intensity profiles from a single image of the swine kidney (corresponding to the red dotted line in Fig. 4c) compared to images compounded at elevational angle of 0° and 5°. As in the case of phantom A (Fig. 2g), the high variation of the A-line intensity profile of the single image across solid tissue sections (blue rectangles in Fig. 4g) is strongly suppressed upon compounding at a 5° elevational angle, but much less so at 0°. It can be seen that electrical noise is largely suppressed using compounding at both 0° and 5° elevational angles, observable in the A-line intensity profile across the hypoechoic regions where minimal speckle source is expected (gray rectangles in Fig. 4g). It is noteworthy that using REACT with 5° elevational compounding shows better efficiency not only in speckle reduction but also in US electrical noise suppression, similar to the results in Fig. 2g. To quantify this observation, Fig. 4h,i show the effect of increasing the number of images used for compounding on SNR, standard deviation (STD), and CNR for 0° and 5° EAC, calculated within the specified ROIs in Fig. 4b. In both cases, SNR and CNR trend upward while STD trends downward, yet with greater rates for the 5° case due to reduction in both speckle and electrical noise.

## Discussion

Speckle noise in US imaging adversely affects its diagnostic accuracy. Among proposed despeckling methods, compounding techniques are preferable because they can improve image quality and reveal real structures obscured by speckle noise, which are otherwise irretrievable using image processing techniques. This functionality requires the acquisition of multiple images with uncorrelated speckle patterns, which often comes at the cost of increased size and complexity of the imaging setup, limiting its applicability for clinical translation. Here, we have demonstrated a new refraction-based elevational angular compounding technique (REACT), which efficiently de-speckles US images using an acoustic refractive element. This method enables EAC in a US system comprising a low-cost 1D detector array without moving or tilting the entire transducer head, eliminating the need for bulky mechanical stages or costly 2D arrays and easing implementation in current US imaging systems equipped with 1D arrays.

In contrast to previous EAC implementations, REACT uses a fixed 1D transducer array with a translating acoustic cylindrical lens. Visual and quantitative inspection of US images depicted in Fig. 2 and Fig. 4 illustrates the capability of REACT to reduce speckle noise. Compounded images acquired with elevational angular deflections of 0°, 2.5°, and 5° improved the CNR by 0.28 × , 1.84 × , and 2.18 × and SNR by 0.23 × , 1.22 × , and 1.72 × compared to single images, respectively. Note that CNR and SNR improvement in the 0° elevational compounded image is due to electrical noise suppression, whereas the corresponding improvement for the 2.5° and 5° cases is due to the both electrical and speckle noise reduction achieved by averaging uncorrelated speckle image patterns from different elevational angular views. As expected, the wider the elevational angular acquisition, the greater the speckle suppression in the compounded image^13,14^. While the widest angle employed in our study was 5°, the selection of the optimal compounding angle should take into account the trade-off between speckle reduction efficiency and image distortion (discussed below). Our results in Table 1 suggest that REACT offers at least twice the enhancement in SNR and CNR compared to previous reported implementations of EAC^14^. This superior enhancement could be attributed to a reduction in motion artefacts because of the fixed transducer. Note that imaging through the PMMA refractive lenses caused a 7.5% signal loss due to acoustic attenuation and reflection (inset in Fig. 2j). REACT’s SNR could be further improved by minimizing this signal loss using materials with lower acoustic impedance and attenuation compared to PMMA (e.g. TPX^27^) and utilizing diffraction lenses with lower effective thicknesses^31^.

Although EAC is recognized as an efficient way to suppress speckle noise in US images, it suffers from anatomic structure deformation and edge blurriness in compounded images captured within wide elevational angular views^13,14^. Our results demonstrate that image deformation can also occur in EAC with narrow elevational angles, particularly from deeper imaging positions, due to the associated increase in elevational angular width (Δ). Image fidelity in EAC, or in other words, accurate representation of structural features from the despeckled plane, is affected by interference from structures in adjacent elevational planes. We explored the trade-off between imaging depth and elevational angular width when employing EAC methods on targets with shapes that have no (cylinder), low (frustum), and high (cone) diameter gradient along the elevational direction (Fig. 3). We found that higher image fidelity is obtained with targets that preserve their cross-sectional appearance along the elevational angular width (cylinder), while image fidelity increasingly suffers for targets with intermediate and high diameter gradients (frustum and cone). The effect of cross-sectional variation on image fidelity was further validated by imaging an *ex vivo* chicken heart and swine kidney with REACT, which demonstrated the importance of accounting for the morphology of the sample for a given elevational angular width in EAC. The spatial resolution along elevational direction is the main limiting factor in all EAC implementation and depends on the total elevational beam width. Hence, out of plane signals can degrade compounding quality, particularly when imaging small organs. This was confirmed in our experiments, where the high variation of the cross-sectional appearance of the chicken heart resulted in distortions in the compounded image (Fig. 3f,g), whereas the low variation in the cross-sectional appearance of the excised swine kidney led to speckle reduction with no loss in image fidelity (Fig. 4b,c). Despeckling of smaller organs using EAC could be performed by reducing the angular compounding width at the cost of a lower despeckling efficiency. We found through experiment that 5° EAC afforded high despeckling efficiency with minimal image fidelity loss in large organs, such as the kidney. However, determination of the optimal elevational angle width for different organs or applications is required to ensure the highest despeckling efficiency without significant loss of image fidelity. Future studies will be conducted on optimization algorithms using quantitative image analysis to investigate the optimal compounding angle for specific targets. REACT could be attractive in targets with lower expected elevational variations such as peripheral vascular, muscle, or bone imaging.

In summary, we utilized acoustic refraction to introduce elevational angular steering into existing 1D transducer arrays and demonstrated its application in a novel implementation of EAC called REACT. Such a low-cost and simple compounding de-speckling method can be of great benefit in clinics to improve current US visualization and interpretation during disease diagnosis. Further work aims at miniaturising ACL embodiments and allowing real-time despeckling by automating the position of the lens and synchronizing frames to evaluate REACT in a clinical context.

## Data Availability

The datasets generated during and/or analysed during the current study are available from the corresponding author on reasonable request.
